# Ischemic colitis related to antipsychotics : A rare and serious entity to know

**DOI:** 10.1192/j.eurpsy.2022.1861

**Published:** 2022-09-01

**Authors:** M. Moalla, M. Moalla, M. Boudabous, N. Tahri

**Affiliations:** Hedi chaker Hospital, Gastroenterology, Sfax, Tunisia

**Keywords:** Ischemic colitis, Side effects, Antipsychotics

## Abstract

**Introduction:**

Ischemic colitis is a rare condition. It represents 3 to 10% of lower digestive hemorrhages. It preferentially affects the subject over the age of 50 with predisposing factors. Rare cases have been reported in young subjects with the use of cocaine, combined hormones or antipsychotics.

**Objectives:**

This work aimed to study the potential side effects of antipsychotics

**Methods:**

We report a case of ischemic colitis associated with antipsychotics.

**Results:**

A 27-year-old patient, followed for 2 years for schizophrenia treated with antipsychotics (chlorpromazine and haloperidol) and an antiparkinsonian (Biperiden), consulted in the emergency room for rectorragies progressing for 3 days. The examination revealed the installation of diffuse abdominal pain associated with early postprandial vomiting which preceded the 7-day rectal bleeding. The physical examination revealed ascites without edema of the lower extremities. The stools were normal-colored on digital rectal examination. The biological workup revealed anemia and a biological inflammatory syndrome. The abdomino-pelvic scanner showed thickening of the entire colonic wall with signs of recent bleeding. The rectosigmoidoscopy showed an ecchymotic aspect of the sigmoid with less pronounced involvement of the rectum. Pathologic examination of the colonic biopsies concluded with ischemic colitis, showing hemorrhagic suffisions with numerous fibrinous thrombi of the vessels. The course was marked by the onset of multi-organ failure with acute renal failure, a picture of disseminated intravascular coagulation (DIC) and alveolar hemorrhage. Despite the resuscitation, the patient died 2 days after admission.

**Conclusions:**

Ischemic colitis is a rare side effect of antipsychotics. Although rare, this entity should be evoked and diagnosed in time.

**Disclosure:**

No significant relationships.

